# Supplementary material for: Environmental variation mediates the evolution of anticipatory parental effects
Source: Evol Lett. 2020 Jun 10;4(4):371–81. doi: 10.1002/evl3.177 (PMC7403678; doi:10.1002/evl3.177)
Supplement: Supplementary file 2 — Supplementary figure 1. Daily reproduction in 25°C when parents are (A) grown for two generations in 20°C or (B) grown for two generations in 25°C. Supplementary figure 2. Daily reproduction in 20°C when parents are also grown for two generations in 20°C. Supplementary table 1. Daily growth factor λ in 25°C. Estimated experimental evolution regime means, with associated 95% confidence limits. Supplementary table 2. Daily growth factor λ in 25°C. Post‐hoc contrasts over temperatures, investigating the significant Regime × Parental temperature interaction from the full model. Supplementary table 3. Total reproduction in 25°C. Estimated experimental evolution regime means, with associated 95% confidence limits. Supplementary table 4. Total reproduction in 25°C. Post‐hoc contrasts over temperatures, investigating the significant Regime × Parental temperature interaction from the full model. Supplementary table 5. Daily growth factor λ in 20°C. Estimated experimental evolution regime means, with associated 95% confidence limits. Supplementary table 6. Daily growth factor λ in 20°C. Post‐hoc contrasts. Supplementary table 7. Total reproduction in 20°C. Estimated experimental evolution regime means, with associated 95% confidence limits. Supplementary table 8. Total reproduction in 20°C. Post‐hoc contrasts. Supplementary table 9. Estimating the effect of Cabinet in the part of the experiment where 2 cabinets per temperature were used (20°C ‐ 20°C and 20°C ‐ 25°C comparisons). Supplementary table 10. Total reproduction. Supplementary table 11. Daily growth factor λ. Supplementary table 12. Daily growth factor λ. [file EVL3-4-371-s002.pdf]

**Supplementary materials**

# Environmental variation mediates the evolution of anticipatory parental effects

Martin I. Lind [1,*], Martyna K. Zwoinska[1], Johan Andersson[1], Hanne Carlsson[1,2], Therese Krieg[1], Tuuli Larva[1] & Alexei A. Maklakov[1,2]

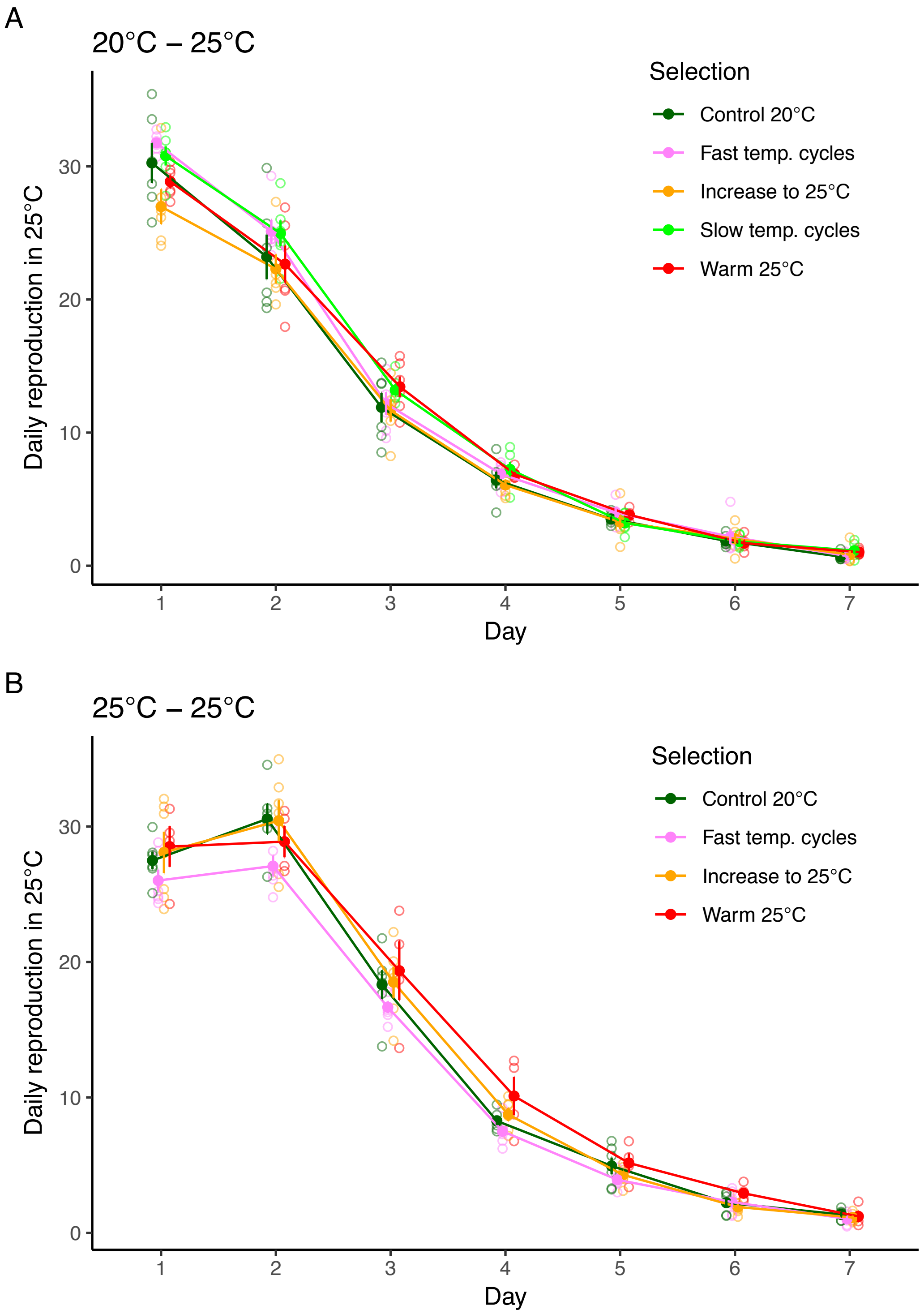


**Supplementary figure 1.** Daily reproduction in 25°C when parents are (A) grown for two generations in 20°C or (B) grown for two generations in 25°C. Symbols represent experimental evolution regime (mean ± SE calculated from line means). Open symbols represent the mean of each replicate line.

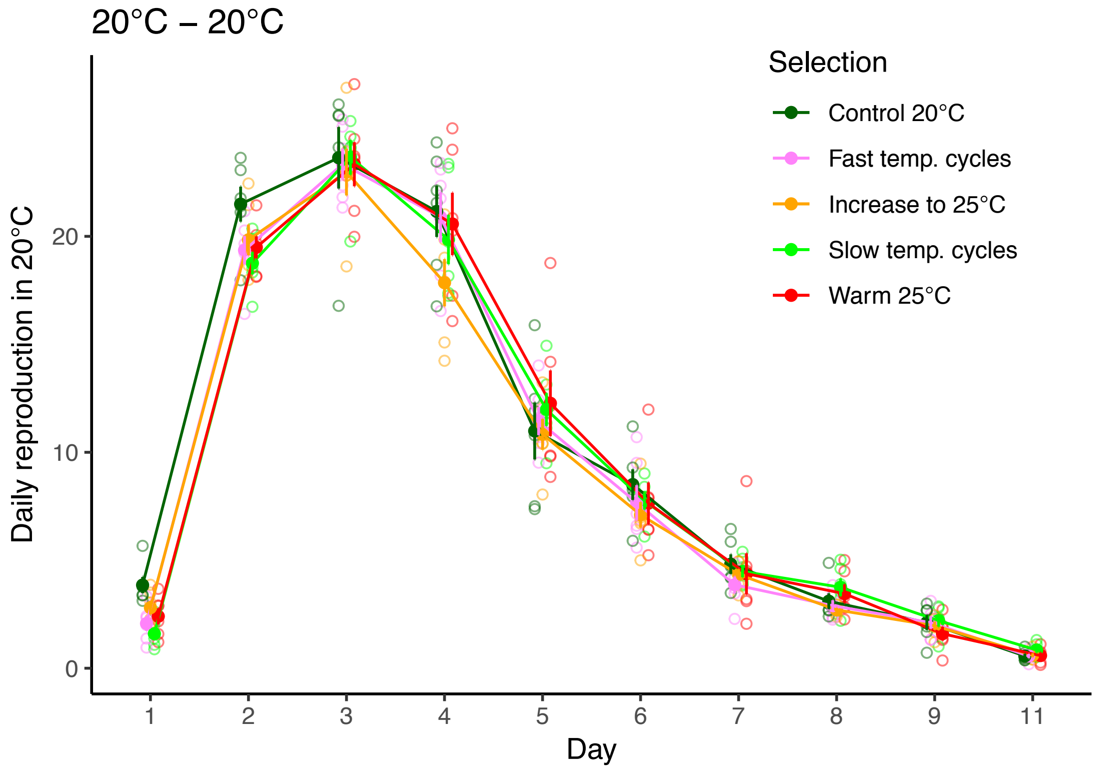


**Supplementary figure 2.** Daily reproduction in 20°C when parents are also grown for two generations in 20°C. Symbols represent experimental evolution regime (mean ± SE calculated from line means). Open symbols represent the mean of each replicate line.

**Supplementary table 1.** Daily growth factor λ in 25°C. Estimated experimental evolution regime means, with associated 95% confidence limits.

| Regime | Parental temp. | emmean | SE | df | Lower CL | Upper CL |
|---|---|---|---|---|---|---|
| Fast temp. cycles | 20°C | 1.23 | 0.0124 | 21.8 | 1.21 | 1.26 |
| Fast temp. cycles | 25°C | 1.19 | 0.0119 | 17 | 1.17 | 1.22 |
| Control 20°C | 20°C | 1.21 | 0.0118 | 18.9 | 1.19 | 1.24 |
| Control 20°C | 25°C | 1.21 | 0.0124 | 18.6 | 1.18 | 1.23 |
| Incr. warming | 20°C | 1.18 | 0.012 | 19.9 | 1.16 | 1.21 |
| Incr. warming | 25°C | 1.22 | 0.0119 | 17 | 1.19 | 1.24 |
| Warm 25°C | 20°C | 1.2 | 0.0118 | 18.9 | 1.18 | 1.23 |
| Warm 25°C | 25°C | 1.22 | 0.0152 | 19.4 | 1.19 | 1.25 |

**Supplementary table 2.** Daily growth factor λ in 25°C. Post-hoc contrasts over temperatures, investigating the significant Regime × Parental temperature interaction from the full model.

| Regime | Contrast | Estimate | SE | df | t-ratio | p-value |
|---|---|---|---|---|---|---|
| Fast temp. cycles | 20°C - 25°C | 0.037 | 0.0151 | 18.8 | 2.481 | 0.023 |
| Control 20°C | 20°C - 25°C | 0.002 | 0.0149 | 18.1 | 0.138 | 0.892 |
| Incr. warming | 20°C - 25°C | -0.036 | 0.0147 | 17.4 | -2.416 | 0.027 |
| Warm 25°C | 20°C - 25°C | -0.015 | 0.0173 | 21.1 | -0.879 | 0.389 |

**Supplementary table 3.** Total reproduction in 25°C. Estimated experimental evolution regime means, with associated 95% confidence limits.

| Regime | Parental temp. | emmean | SE | df | Lower CL | Upper CL |
|---|---|---|---|---|---|---|
| Fast temp. cycles | 20°C | 4.4 | 0.045 | 21.8 | 4.31 | 4.5 |
| Fast temp. cycles | 25°C | 4.43 | 0.044 | 17 | 4.34 | 4.53 |
| Control 20°C | 20°C | 4.34 | 0.042 | 18.9 | 4.25 | 4.43 |
| Control 20°C | 25°C | 4.51 | 0.045 | 18.6 | 4.42 | 4.61 |
| Incr. warming | 20°C | 4.27 | 0.043 | 19.9 | 4.18 | 4.36 |
| Incr. warming | 25°C | 4.52 | 0.044 | 17 | 4.42 | 4.61 |
| Warm 25°C | 20°C | 4.35 | 0.042 | 18.9 | 4.26 | 4.44 |
| Warm 25°C | 25°C | 4.55 | 0.055 | 19.6 | 4.43 | 4.67 |

**Supplementary table 4.** Total reproduction in 25°C. Post-hoc contrasts over temperatures, investigating the significant Regime × Parental temperature interaction from the full model.

| Regime | Contrast | Estimate | SE | df | t-ratio | p-value |
|---|---|---|---|---|---|---|
| Fast temp. cycles | 20°C - 25°C | -0.030 | 0.0529 | 18.7 | -0.56 | 0.582 |
| Control 20°C | 20°C - 25°C | -0.177 | 0.0524 | 18 | -3.378 | 0.003 |
| Incr. warming | 20°C - 25°C | -0.243 | 0.0516 | 17.3 | -4.699 | <0.001 |
| Warm 25°C | 20°C - 25°C | -0.200 | 0.0613 | 20.8 | -3.263 | 0.004 |

**Supplementary table 5.** Daily growth factor λ in 20°C. Estimated experimental evolution regime means, with associated 95% confidence limits.

| Selection | emmean | SE | df | Lower CL | Upper CL |
|---|---|---|---|---|---|
| Control 20°C | 0.95 | 0.00963 | 22.4 | 0.93 | 0.97 |
| Fast temp. cycles | 0.909 | 0.00923 | 19.1 | 0.89 | 0.928 |
| Incr. warming | 0.92 | 0.00943 | 20.6 | 0.901 | 0.94 |
| Slow temp. cycles | 0.899 | 0.00923 | 19.1 | 0.879 | 0.918 |
| Warm 25°C | 0.916 | 0.00932 | 19.8 | 0.897 | 0.936 |

**Supplementary table 6.** Daily growth factor λ in 20°C. Post-hoc contrasts.

| Contrast | Estimate | SE | df | t-ratio | p-value |
|---|---|---|---|---|---|
| Control - Fast | 0.04118 | 0.0122 | 25.8 | 3.383 | 0.0178 |
| Control - Incr. | 0.02982 | 0.0123 | 26.9 | 2.421 | 0.1403 |
| Control - Slow | 0.05137 | 0.0122 | 25.8 | 4.22 | 0.0023 |
| Control - Warm | 0.03386 | 0.0122 | 26.3 | 2.764 | 0.0708 |
| Fast - Incr. | -0.01136 | 0.012 | 24.5 | -0.946 | 0.8761 |
| Fast - Slow | 0.01019 | 0.0119 | 23.4 | 0.86 | 0.9086 |
| Fast - Warm | -0.00733 | 0.0119 | 23.9 | -0.614 | 0.9714 |
| Incr. - Slow | 0.02155 | 0.012 | 24.5 | 1.794 | 0.3995 |
| Incr. - Warm | 0.00404 | 0.0121 | 25 | 0.334 | 0.9971 |
| Slow - Warm | -0.01752 | 0.0119 | 23.9 | -1.468 | 0.5917 |

**Supplementary table 7.** Total reproduction in 20°C. Estimated experimental evolution regime means, with associated 95% confidence limits.

| Selection | emmean | SE | df | Lower CL | Upper CL |
|---|---|---|---|---|---|
| Control 20°C | 4.6 | 0.0477 | 26.4 | 4.5 | 4.7 |
| Fast temp. cycles | 4.54 | 0.0462 | 23.4 | 4.44 | 4.63 |
| Incr. warming | 4.5 | 0.047 | 24.7 | 4.41 | 4.6 |
| Slow temp. cycles | 4.54 | 0.0462 | 23.4 | 4.45 | 4.64 |
| Warm 25°C | 4.55 | 0.0466 | 24 | 4.45 | 4.65 |

**Supplementary table 8.** Total reproduction in 20°C. Post-hoc contrasts.

| Contrast | Estimate | SE | df | t-ratio | p-value |
|---|---|---|---|---|---|
| Control - Fast | 0.06624 | 0.0661 | 25.5 | 1.002 | 0.852 |
| Control - Incr. | 0.09864 | 0.0666 | 26.2 | 1.481 | 0.5832 |
| Control - Slow | 0.06132 | 0.0661 | 25.5 | 0.928 | 0.8836 |
| Control - Warm | 0.05202 | 0.0664 | 25.9 | 0.784 | 0.933 |
| Fast - Incr. | 0.0324 | 0.0656 | 24.7 | 0.494 | 0.9872 |
| Fast - Slow | -0.00493 | 0.065 | 24 | -0.076 | 1 |
| Fast - Warm | -0.01422 | 0.0653 | 24.3 | -0.218 | 0.9995 |
| Incr. - Slow | -0.03732 | 0.0656 | 24.7 | -0.569 | 0.9784 |
| Incr. - Warm | -0.04661 | 0.0659 | 25 | -0.708 | 0.9528 |
| Slow - Warm | -0.00929 | 0.0653 | 24.3 | -0.142 | 0.9999 |

## Supplementary analysis 1

### The effect of cabinet

***Methods***
Although most of the experiment (the 20°C - 20°C and 20°C - 25°C assays) used two replicate cabinets per temperature, part of the experiment (the 25°C - 25°C assays) only used a single cabinet per temperature. Therefore, we further investigated the effect of cabinet, in order to ensure that the effect of temperature is not influenced by whether cabinet is included in the model or not. Cabinet cannot be fitted as a fixed effect, since the same cabinets were not used for both temperatures (two in 20°C, two other in 25°C).

We therefore analysed the effect of cabinet in the 20°C - 20°C and 20°C - 25°C assays, by fitting it as a random effect, alternatively by excluding it from the model. Response variables we either total reproduction or daily growth factor λ, both were log transformed. As fixed effects, we fitted Selection regime, Temperature treatment, and their interaction. As random effect, we fitted replicate population, and we either included or omitted the random effect of Cabinet. Models were constructed with a random slope (parental temperature) in addition to random intercept.

All statistical analyses were done using *R 3.6.1*, and models were implemented using the *lme4* package (Bates et al. 2015). Significance tests were performed using the *lmerTest* package (Kuznetsova et al. 2017). However, due to singularity in the estimation of the Population effect in the Daily growth factor λ analyses (which can overestimate the degrees of freedom in the ML/REML models), these analyses were also repeated in a Bayesian framework in the *rstanarm* package (Goodrich et al. 2020) using default priors.

***Results***
For total reproduction, we found that the best model is without the random effect of cabinet (Supplementary table 9), The significance of the parameters remains unaltered depending upon model, temperature having a significant effect in both models (Supplementary table 10).

For λ, we run into singularity problems (when estimating the Population effect) using ML/REML estimation, therefore we also estimated this model in a Bayesian framework using the *rstanarm* package. While the LMER approach suggests that the best model is without Cabinet, the Bayesian approach suggests that the best model incorporates cabinet, but the difference between these models is smaller than the standard error of the difference, indicating no significant improvement of including cabinet. Moreover, the estimation of the fixed effect components is almost identical between the models within each approach Supplementary table 11-12), and also between the REML and Bayesian approach. In both cases, temperature has a strong effect on λ, and the inclusion or removal of Cabinet does not influence the estimation of any fixed parameters.

Based upon these analyses, we conclude that temperature, rather than cabinet, is driving the observed patterns.

**Supplementary table 9.** Estimating the effect of Cabinet in the part of the experiment where 2 cabinets per temperature were used (20°C - 20°C and 20°C - 25°C comparisons). Bayesian models are compared using ELPD difference, where the best model is indicated by "0" (large ELPD is best).

| **Response** | **Cabinet in model** | **Model type** | **AIC** | **ELPD difference (+ SE)** |
|---|---|---|---|---|
| Total reproduction | Yes | ML | -187.37 | - |
| Total reproduction | - | ML | -189.34 | - |
| Daily growth factor λ | Yes | ML | -823.79 | - |
| Daily growth factor λ | - | ML | -825.35 | - |
| Daily growth factor λ | Yes | Bayesian | - | 0.0 + 0.0 |
| Daily growth factor λ | - | Bayesian | - | -0.6 + 2.0 |

**Supplementary table 10. Total reproduction**. The effect of selection regime and temperature treatment on total reproduction (parents in 20°C, offspring in 20°C or 25°C). Models with and without cabinet as a random effect, in order to estimate if inclusion or removal of cabinet change the parameter estimates.

| | **Cabinet in model** | | | | | **Without cabinet in model** | | | | |
|---|---|---|---|---|---|---|---|---|---|---|
| **Fixed coefficient** | **Estimate** | **SE** | **df** | **t** | **p** | **Estimate** | **SE** | **df** | **t** | **p** |
| Intercept (*Control 20°C*) | 4.603 | 0.050 | 23.5 | 92.17 | <0.001 | 4.603 | 0.048 | 27.4 | 95.79 | <0.001 |
| *Fast temp. cycles* | -0.066 | 0.067 | 25.3 | -0.99 | 0.333 | -0.066 | 0.067 | 25.4 | -0.99 | 0.330 |
| *Incr. warming* | -0.097 | 0.068 | 26.2 | -1.43 | 0.165 | -0.097 | 0.067 | 26.3 | -1.45 | 0.159 |
| *Slow temp. cycles* | -0.061 | 0.067 | 25.3 | -0.91 | 0.370 | -0.061 | 0.067 | 25.4 | -0.92 | 0.367 |
| *Warm 25°C* | -0.051 | 0.067 | 25.7 | -0.76 | 0.454 | -0.051 | 0.067 | 25.8 | -0.76 | 0.456 |
| Temp. treatment 25°C | -0.265 | 0.053 | 17.5 | -5.00 | <0.001 | -0.265 | 0.050 | 26.6 | -5.30 | <0.001 |
| *Fast temp. cycles* × Temp. 25°C | 0.130 | 0.070 | 26.0 | 1.85 | 0.075 | 0.131 | 0.070 | 26.0 | 1.86 | 0.075 |
| *Inc. warming* × Temp. 25°C | 0.033 | 0.070 | 25.9 | 0.47 | 0.643 | 0.034 | 0.070 | 25.9 | 0.49 | 0.631 |
| *Slow temp. cycles* × Temp. 25°C | 0.126 | 0.069 | 24.8 | 1.82 | 0.081 | 0.126 | 0.069 | 24.8 | 1.82 | 0.082 |
| *Warm 25°C* × Temp. 25°C | 0.064 | 0.070 | 25.2 | 0.92 | 0.365 | 0.064 | 0.070 | 25.2 | 0.91 | 0.370 |

**Supplementary table 11. Daily growth factor λ.** The effect of selection regime and temperature treatment on daily growth factor λ (parents in 20°C, offspring in 20°C or 25°C). Models with and without cabinet as a random effect, in order to estimate if inclusion or removal of cabinet change the parameter estimates. Singularity in model overestimates the degrees of freedom, se Supplementary table 12 for implementation in a Bayesian framework.

| | Cabinet in model | | | | | Without cabinet in model | | | | |
|---|---|---|---|---|---|---|---|---|---|---|
| **Fixed coefficient** | **Estimate** | **SE** | **df** | **t** | **p** | **Estimate** | **SE** | **df** | **t** | **p** |
| Intercept (*Control 20°C*) | 0.950 | 0.009 | 13.9 | 102.21 | <0.001 | 0.950 | 0.008 | 187.3 | 116.19 | <0.001 |
| *Fast temp. cycles* | -0.041 | 0.011 | 189.2 | -3.75 | <0.001 | -0.041 | 0.011 | 187.3 | -3.72 | <0.001 |
| *Incr. warming* | -0.030 | 0.011 | 189.3 | -2.69 | 0.008 | -0.030 | 0.011 | 187.3 | -2.70 | 0.008 |
| *Slow temp. cycles* | -0.051 | 0.011 | 189.2 | -4.68 | <0.001 | -0.051 | 0.011 | 187.3 | -4.64 | <0.001 |
| *Warm 25°C* | -0.034 | 0.011 | 189.2 | -3.06 | 0.003 | -0.034 | 0.011 | 187.3 | -3.01 | 0.003 |
| Temp. treatment 25°C | 0.261 | 0.015 | 19.2 | 17.29 | <0.001 | 0.261 | 0.014 | 44.3 | 19.05 | <0.001 |
| *Fast temp. cycles* × Temp. 25°C | 0.061 | 0.019 | 52.5 | 3.19 | 0.002 | 0.062 | 0.019 | 43.6 | 3.19 | 0.003 |
| *Inc. warming* × Temp. 25°C | -0.001 | 0.019 | 52.5 | -0.05 | 0.959 | 0.000 | 0.019 | 43.5 | -0.02 | 0.981 |
| *Slow temp. cycles* × Temp. 25°C | 0.064 | 0.019 | 50.7 | 3.36 | 0.001 | 0.064 | 0.019 | 42.0 | 3.35 | 0.002 |
| *Warm 25°C* × Temp. 25°C | 0.025 | 0.019 | 51.3 | 1.33 | 0.189 | 0.025 | 0.019 | 42.5 | 1.32 | 0.194 |

**Supplementary table 12. Daily growth factor λ.** The effect of selection regime and temperature treatment on daily growth factor λ (parents in 20°C, offspring in 20°C or 25°C). Models with and without cabinet as a random effect, in order to estimate if inclusion or removal of cabinet change the parameter estimates. Model implemented in a Bayesian framework. The 95% confidence interval (2.5% - 97.5%) is considered significantly different from zero if it does not include zero.

| | **Cabinet in model** | | | | | **Without cabinet in model** | | | | |
|---|---|---|---|---|---|---|---|---|---|---|
| | **Estimate** | **SD** | **2.5%** | **97.5%** | **Significant** | **Estimate** | **SD** | **2.5%** | **97.5%** | **Significant** |
| Intercept (*Control 20°C*) | 0.949 | 0.014 | 0.921 | 0.976 | Yes | 0.950 | 0.010 | 0.930 | 0.970 | Yes |
| *Fast temp. cycles* | -0.040 | 0.013 | -0.067 | -0.014 | Yes | -0.041 | 0.013 | -0.067 | -0.014 | Yes |
| *Incr. warming* | -0.029 | 0.013 | -0.055 | -0.002 | Yes | -0.030 | 0.014 | -0.056 | -0.003 | Yes |
| *Slow temp. cycles* | -0.051 | 0.013 | -0.078 | -0.024 | Yes | -0.051 | 0.014 | -0.078 | -0.024 | Yes |
| *Warm 25°C* | -0.034 | 0.013 | -0.059 | -0.007 | Yes | -0.033 | 0.014 | -0.061 | -0.007 | Yes |
| Temp. treatment 25°C | 0.262 | 0.019 | 0.224 | 0.302 | Yes | 0.261 | 0.012 | 0.236 | 0.286 | Yes |
| *Fast temp. cycles* × Temp. 25°C | 0.061 | 0.017 | 0.027 | 0.094 | Yes | 0.061 | 0.018 | 0.027 | 0.096 | Yes |
| *Inc. warming* × Temp. 25°C | -0.002 | 0.017 | -0.035 | 0.033 | - | -0.001 | 0.018 | -0.035 | 0.033 | - |
| *Slow temp. cycles* × Temp. 25°C | 0.063 | 0.017 | 0.030 | 0.095 | Yes | 0.064 | 0.017 | 0.030 | 0.097 | Yes |
| *Warm 25°C* × Temp. 25°C | 0.025 | 0.017 | -0.007 | 0.058 | - | 0.025 | 0.017 | -0.008 | 0.060 | - |
